# Measurements of Temperature Distribution for High Temperature Steel Plates Based on Digital Image Correlation

**DOI:** 10.3390/ma12203322

**Published:** 2019-10-12

**Authors:** Pengyuan Qi, Gang Wang, Zhen Gao, Xianghua Liu, Weijie Liu

**Affiliations:** 1 School of Materials Science and Engineering, Northeastern University, Shenyang 110819, China; qipengyuan@126.com (P.Q.); waynejliu@hotmail.com (W.L.); 2 Department of Materials Science and Engineering, Yingkou Institute of Technology, Yingkou 115014, China; dagang6@aliyun.com (G.W.); gz860310@126.com (Z.G.)

**Keywords:** image processing, temperature measurement, radiant heat, non-contact

## Abstract

Temperature distribution is an important process parameter of steel plates during electromagnetic induction heating treatment. This study uses the digital image correlation method to develop an effective non-contact temperature measurement that allows obtaining valuable information about the temperature value of a high temperature steel plate specimen and analyzing its temperature distribution. A principle of thermal radiation temperature measurement based on the color chagre couled device (CCD) technology was introduced. The image processing system encapsulates the image update module, form mode module, image event module and temperature analysis module. The error analysis and temperature calibration were carried out to make sure the error deviation of the measurement system was within a small range. The temperature distribution of B1500HS at high temperature was analyzed by the designed measurement system which was in good agreement with the result from Raynger 3i Plus temperature gun, indicating that the measurement system based on image processing basically meets the requirements of temperature distribution measurement of a high temperature steel plate, and provides an important reference for a high temperature steel plate in non-contact temperature distribution measurement.

## 1. Introduction

So far, the most widely used method of measuring temperature in the heat treatment industry is the thermal couple system, however, the traditional contact temperature measurement with thermocouples cannot reflect the overall temperature distribution of the heated workpiece. Besides, the thermocouple will self-heat and affect workpiece temperature conduction in electromagnetic induction heating treatment [1,2,3]. 

Digital image correlation (DIC) can realize non-contact and full-field analysis and provide more data in-situ that are not possible with traditional techniques. Thus, this technique is widely applied in materials’ mechanical behaviors and properties research at high temperature. Herrera-Solaz et al. [4] used DIC technique to track the strain maps during tension loading in order to compare the local strain fields on the microscopic scale of an austenitic stainless steel 316L. Dinh Ba Le et al. [5] applied a DIC system for reliable deformation measurement of concrete structures at high temperature. Jung et al. [6] investigated the mesoscopic deformation behavior of open-cell metal/polymer hybrid foams by using correlative DIC and infrared thermography measurements. IR thermography equipment is usually composed of optical system, spectral filtering, infrared detector array, video image processing and so on [7]. Because of the wide range of infrared radiation, the IR thermography equipment can measure the temperature in a wide range [8], but the structure of IR thermography equipment is quite complex and expensive. 

In the heat treatment process of a steel plate, the infrared radiation energy of the workpiece changes in a specific range and the temperature of a thermal radiator can be visually measured by its color and brightness. When the temperature of the radiator is measured by a color CCD camera, the amount of radiation of the object can be converted into a corresponding luminous flux by the imaging device, and then visualized by an optical image. Thus, temperature calibration [9,10,11,12,13] is performed on the digital signal to analyze the relationship between the object radiation temperature and the optical channel intensity value obtained by the color CCD camera.

**Figure 1:**
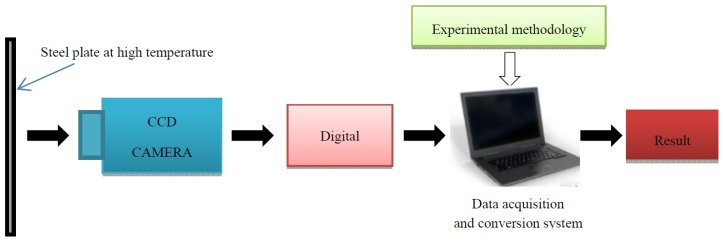
Diagram of temperature field measurement system for a high temperature steel plate.

In this paper, on the basis of heat treatment process of a steel plate by electromagnetic induction heating, a special system for high temperature measurement based on the digital image correlation has been developed, as shown in Figure 1. The system uses a new type of radiation temperature measurement method to correlate the color and brightness of high-temperature steel plates with temperature and combines modern digital image correlation technology with traditional thermal radiation theory to provide non-contact temperature measurement based on color digital images. The temperature measurement system in this paper consists of simple and practical detection equipment, and the cost is much lower than that of IR thermography equipment. The system was tested through practical application; the temperature field distribution of the B1500HS boron steel plate during high temperature heating was obtained by this system, and the results meet the requirements.

## 2. Principle of Thermal Radiation Temperature Measurement

The basic theory of heat transfer shows that any object that exceeds absolute zero emits thermal radiation [14,15,16]. The radiation temperature measurement method is based on the thermal radiation information emitted from the surface of the measuring object and is measured by Planck’s law. Planck’s law states that the energy M(*λ*,*T*) (i.e., the spectral radiation) is radiated from the unit surface area per unit time in all directions of the hemispherical space. It can be calculated according to Equation (1).

(1) $$M(\lambda ,T)=\frac{\varepsilon (\lambda ,T)C_{1}\lambda^{-5}}{e^{C_{2}/(\lambda ,T)}-1},$$

where *λ* is the wavelength (m), *T* is the thermodynamic temperature (K) of the measured object, *ε*(*λ*,*T*) is the surface emissivity of the measured object, *C_1_* is the first radiating constant (3.7419 × 10^−16^ W·m^2^) and *C_2_* is the second emission constant (1.4388 × 10^−2^ m·K). It can be seen from Equation (1) that Planck’s law describes the law that the radiant energy of the measured object is distributed according to the wavelength.

Since the radiation wavelength is less than 1000 nm and the temperature is less than 3000 K, Equation (1) can be replaced by Wayne’s law of radiation.

(2) $$M(\lambda ,T)=\varepsilon (\lambda ,T)\frac{C_{1}}{\lambda^{5}}\exp(-\frac{C_{2}}{\lambda T})$$

The emitted monochromatic radiant energy values M(*λ_1_*,*T*), M(λ_2_,*T*) and M(*λ_3_*,*T*) are measured by the wavelengths of λ_1_, λ_2_ and λ_3_, respectively, and obtain Equation (3).

(3) $$\frac{M(\lambda_{1},T)\cdot M(\lambda_{3},T)}{M^{2}(\lambda_{2},T)}=\frac{\varepsilon (\lambda_{1},T)\cdot \varepsilon (\lambda_{3},T)}{\varepsilon^{2}(\lambda_{2},T)}(\frac{\lambda_{2}^{2}}{\lambda_{1}\lambda_{3}})\exp\left[\frac{C_{2}}{T}(\frac{2}{\lambda_{2}}-\frac{1}{\lambda_{1}}-\frac{1}{\lambda_{3}})\right]$$

Then, Equation (4) can be deduced.

(4) $$T=\frac{C_{2}(\frac{2}{\lambda_{2}}-\frac{1}{\lambda_{1}}-\frac{1}{\lambda_{3}})}{\ln\left[\frac{M(\lambda_{1},T)\cdot M(\lambda_{3},T)}{M^{2}(\lambda_{2},T)}\right]-\ln\left[\frac{\varepsilon^{2}(\lambda_{2},T)}{\varepsilon (\lambda_{1},T)\cdot \varepsilon (\lambda_{3},T)}\right]-5\ln(\frac{\lambda_{2}^{2}}{\lambda_{1}\lambda_{3}})}$$

The high temperature steel plate thermal radiation photo is imaged on the CCD through an optical system. In order to perform computer processing, the image signal should be discretized into a digital signal, and the image is digitized and decomposed into a red component intensity R, a green component intensity G, and a blue component intensity B [17,18,19,20,21]. At the end of the camera, the ordinary single-tube color CCD camera obtains three chrominance signals, R_e_, G_e_, and B_e_, in the visible range by means of three kinds of stripe filters on the target surface of the array’s photoelectric element. In a certain test distance and optical system, if the object radiance spectrum is M(*λ*,*T*), the output of the three channels can be deduced from Equation (5).

(5) $$\left\{ \begin{array}{l} R_{e}=k_{0}\int_{320}^{720}M(\lambda ,T)\cdot K_{R}(\lambda )d\lambda \\ G_{e}=k_{0}\int_{320}^{720}M(\lambda ,T)\cdot K_{G}(\lambda )d\lambda \\ B_{e}=k_{0}\int_{320}^{720}M(\lambda ,T)\cdot K_{B}(\lambda )d\lambda \end{array}\right.$$

**Figure 2:**
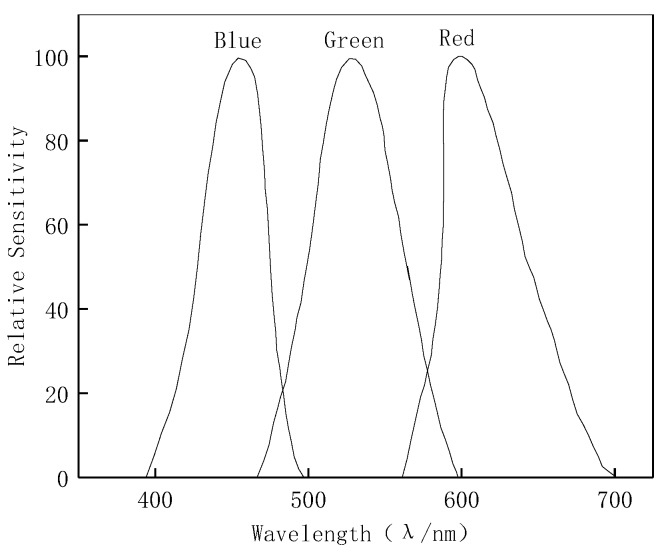
Spectral characteristics of the camera.

In order to calibrate the measurement errors, including the changes in illumination of the environment, loss and attenuation of radiation energy, and the effect of specific optical systems, the system gain parameter *k_0_* and the spectral response functions K_x_(*λ*), x = (R, G, B) are introduced to reflect the characteristics of measurement system. $k_{0}=\frac{1}{4}\tau \eta t{\left(\frac{D}{f}\right)}^{2}$, where *τ* is the transmission coefficient of optical system, *η* is the photoelectric conversion coefficient of CCD, *t* is the exposure time and $\frac{D}{f}$ is the relative aperture; the above parameters can be set up in a digital camera device to achieve the calibration of illumination. K_x_(*λ*), x = (R, G, B) are the spectral response functions which can be derived from the calibration experiment described in the section below to calibrate the performance of three channels of R, G and B. λ_R_, λ_G_ and λ_B_ denote the wavelengths corresponding to the peak values of the spectral characteristic curves of the three channels of R, G and B, respectively, of the acquired image. According to ISO/CIE 11664 Standard, the values of λ_R_, λ_G_ and λ_B_ are 700 nm, 546.1 nm and 435.8 nm, respectively [22,23], and their characteristic curves are similar to those shown in Figure 2.

The three primary color temperature measurement equation is as follows:

(6) $$T=\frac{\frac{a_{1}}{2}\cdot C_{2}(\frac{1}{\lambda_{G}}-\frac{1}{\lambda_{R}})+\frac{a_{2}}{2}\cdot C_{2}(\frac{1}{\lambda_{B}}-\frac{1}{\lambda_{G}})-1}{\frac{a_{1}}{2}\cdot \left\{\ln\left[\frac{R_{e}(T)}{G_{e}(T)}\right]+5\ln(\frac{\lambda_{R}}{\lambda_{G}})\right\}+\frac{a_{2}}{2}\cdot \left\{\ln\left[\frac{G_{e}(T)}{B_{e}(T)}\right]+5\ln(\frac{\lambda_{G}}{\lambda_{B}})\right\}-\frac{b_{1}+b_{2}}{2}},$$

where a_1,_ a_2_, b_1_, and b_2_ are coefficients of temperature calibration from Equations (7) and (8).

## 3. Image Processing System

The temperature measurement image processing system uses Microsoft Visual Studio (referred to as VS) to run the form program on the Windows system platform and uses the C# programming language to write and implement. The system structure and module are as follows.

**Figure 3:**
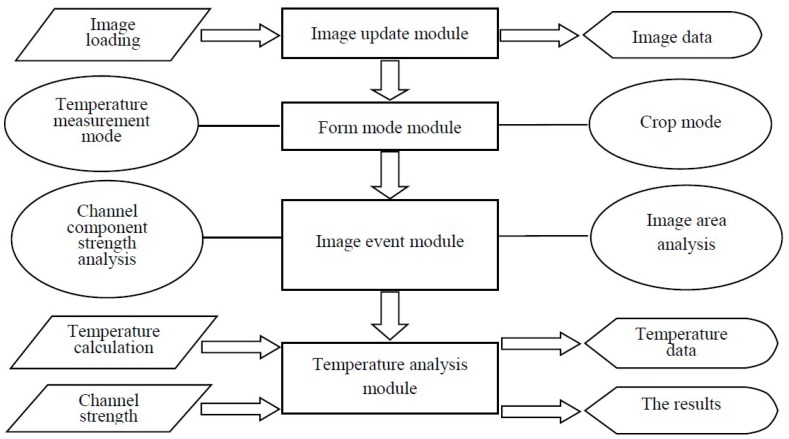
Structure of system function.

Figure 3 shows the functional structure diagram of the system, which mainly includes the following modules: image update module, form mode module, image event module and temperature calculation module.

### 3.1. Image Update Module

The CCD extracted image can be converted into a digital signal, but this may lead to more noise. Median filtering is a nonlinear smoothing technique, which uses the median value of the gray value of all pixels in the neighborhood window of each point as the gray value of each pixel. It has a good effect on removing image noise. It is especially useful for speckle noise and salt and pepper noise. Thus, this module uses a square filter window to capture images, and the steps are as follows:

1. The captured pictures are written to the memory in the format of a bitmap and the picture is displayed.
2. Input of image data is realized and stored in an external memory; output is browsed.
3. The picture size is automatically adjusted to fit the window output.

### 3.2. Form Mode Module

1. Complete the selection of the temperature detection mode or the picture cropping mode.
2. Select the temperature detection mode and perform temperature measurement on the entire window image.
3. Select the picture cropping mode and cut the window image to analyze the area for temperature analysis.

### 3.3. Image Event Module

1. Complete the image update display of the frame selection area.
2. Digitize the image area by pixels.
3. Traverse the area pixels, record the number of occurrences of the color component intensity values of each channel and store them in an array.

### 3.4. Temperature Analysis Module

The color component intensity value array is traversed to calculate the temperature average, the highest temperature value, the lowest temperature value and the highest temperature point coordinate in the image region.

## 4. Temperature Calibration and Results Analysis

### 4.1. Error Analysis

#### 4.1.1. CCD Halo

Due to the characteristics of CCD devices, measurement errors will be introduced. Halo is one of the major factors affecting the acquisition of high quality images by CCD [24]. When the number of photoelectrons generated under strong light exceeds the maximum number of electrons that can be stored in the charge storage area of the CCD, the overflowed electrons will enter the adjacent pixels along the row or column directions, and the halo effect will appear in the accessory area of the bright spot light source [25]. The flare will cause the image sharpness to decrease significantly, and it cannot reflect the details of the area to be observed. In the experiment, the device adopted reducing aperture and an installed pre-attenuator to reduce the brightness of the image reaching the target surface of CCD, so as to avoid halo generation [26]. 

#### 4.1.2. Acquisition Distance

**Figure 4:**
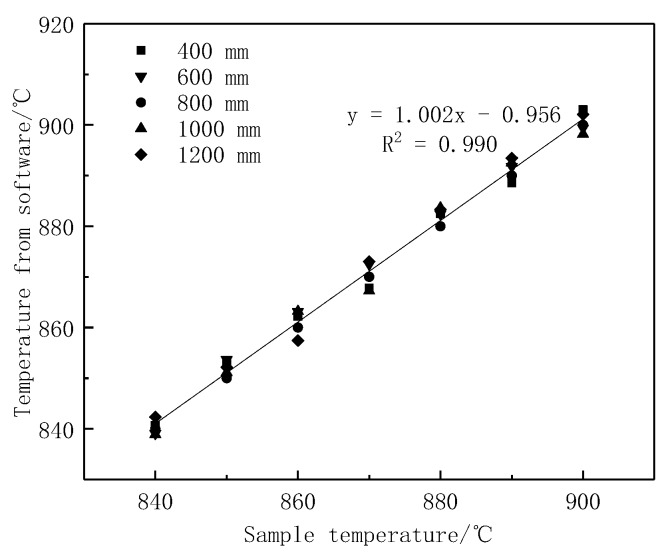
Error analysis of distance change.

In order to study the influence of image acquisition distance on the temperature measurement error, the temperature measurement experiments were carried out at distances of 400, 600, 1000 and 1200 mm from the sample, and compared with the distance of 800 mm at the initial temperature measurement. The experimental results were shown in Figure 4 and Table 1. The analysis showed that the distance change produced an absolute deviation within 3.6 °C and caused a standard deviation within 2.6 °C.

### 4.2. Temperature Calibration

#### 4.2.1. Calibration Experiment

**Figure 5:**
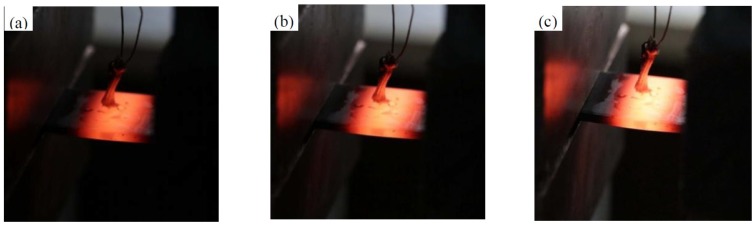
The heated steel plate sample at the same temperature under speed of (**a**) ISO-1600; (**b**) ISO-3200; and (**c**) ISO-6400.

In order to guarantee the accuracy of the measurement, the test needed to be carried out in a dark and closed room as the standard test environment, which is similar to the test condition of blackbody radiation experiments. The collection of calibration database images was completed on the MS-300 thermomechanical simulator. The temperature of the steel plate sample was measured by a thermocouple welded at the center of the steel plate in the temperature range of 650–950 °C. The temperature of B1500HS boron steel plate was measured once every 10 °C interval with a Canon EOS 80D camera. At 800 mm away from the steel plate sample, the thermal radiation images of B1500HS boron steel plate at different temperature were taken with the speeds of ISO-1600, ISO-3200 and ISO-6400. For example, Figure 5 shows the images of steel plate samples obtained at the temperature of 900 °C. In the same way, all the standard images of high temperature steel plates were obtained from the collected images. Finally, the software calibration database was established by using the standard images [27,28,29].

#### 4.2.2. Image Processing Analysis

**Figure 6:**
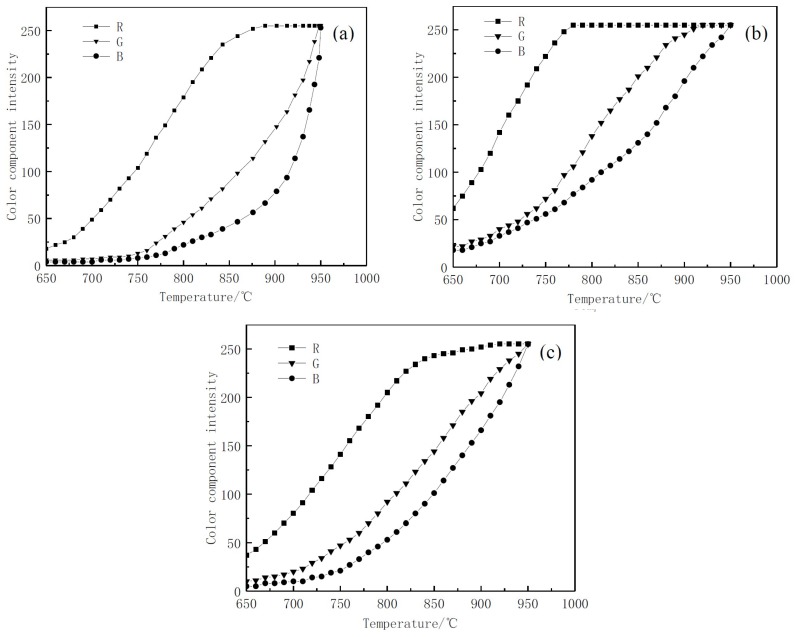
Curve of color component intensity versus temperature under speeds of (**a**) ISO-1600; (**b**) ISO-6400; and (**c**) ISO-3200.

After the image was digitally processed by the computer, it could be decomposed into three image channels of red, green and blue, which were represented by red component intensity R, green component intensity G and blue component intensity B, respectively. Under different sensitivities, the relationship between the intensity of the color component of sample and the heated temperature is shown in Figure 6. 

The relationship between the color component intensity and temperature of the high temperature steel plate image under speeds of ISO-1600 and ISO-6400 is shown in Figure 6a,b. It can be seen that due to the short exposure time of ISO-1600, the brightness of the image was too low, the temperature reaction below 750 °C was not sensitive and the slope was too large at around 930 °C, resulting in the actual temperature measurement effect not being ideal. When ISO-6400 exposure level was used for calibration, due to the long exposure time, the pixels in the image were saturated quickly, resulting in a shorter range. The R value reaches the saturation value of 255 at about 775 °C. As a result, the subsequent values could not reflect the change of the temperature and the actual range was too small.

The relationship between the color component intensity of the steel plate image and the temperature under sensitivity ISO-3200 is shown in Figure 6c. It shows the R, G and B values all had wide valid ranges and high calibration accuracy. So the speed of ISO-3200 was finally used for temperature calibration. According to the R, G and B wavelengths from ISO-3200 database, the relationship between the intensity values of the color components $\ln(\frac{K_{G}}{K_{R}})$ and the temperature T was deduced in Equation (7), as was the relationship between $\ln(\frac{K_{B}}{K_{G}})$ and T in Equation (8).

(7) $$\ln(\frac{K_{G}}{K_{R}})=C_{2}(\frac{1}{\lambda_{G}}-\frac{1}{\lambda_{R}})\frac{1}{T}-\ln\left[\frac{R_{e}(T)}{G_{e}(T)}\right]-5\ln(\frac{\lambda_{R}}{\lambda_{G}})$$

(8) $$\ln(\frac{K_{B}}{K_{G}})=C_{2}(\frac{1}{\lambda_{B}}-\frac{1}{\lambda_{G}})\frac{1}{T}-\ln\left[\frac{G_{e}(T)}{B_{e}(T)}\right]-5\ln(\frac{\lambda_{G}}{\lambda_{B}})$$

Then, the coefficients in Equation (6) such as $a_{1}=\frac{\lambda_{R}\;\lambda_{G}}{C_{2}\;(\lambda_{R}-\lambda_{G})}$, $a_{2}=\frac{\lambda_{B}\;\lambda_{G}}{C_{2}\;(\lambda_{G}-\lambda_{B})}$, $b_{1}=\frac{\lambda_{R}\;\lambda_{G}}{C_{2}\;(\lambda_{R}-\lambda_{G})}\;\left\{\ln\left[\frac{R_{e}(T)}{G_{e}(T)}\right]+5\ln(\frac{\lambda_{R}}{\lambda_{G}})\right\}$ and $b_{2}=\frac{\lambda_{B}\;\lambda_{G}}{C_{2}\;(\lambda_{G}-\lambda_{B})}\;\left\{\ln\left[\frac{G_{e}(T)}{B_{e}(T)}\right]+5\ln(\frac{\lambda_{G}}{\lambda_{B}})\right\}$ were deduced from Equations (7) and (8). 

### 4.3. Experimental Test Analysis

**Figure 7:**
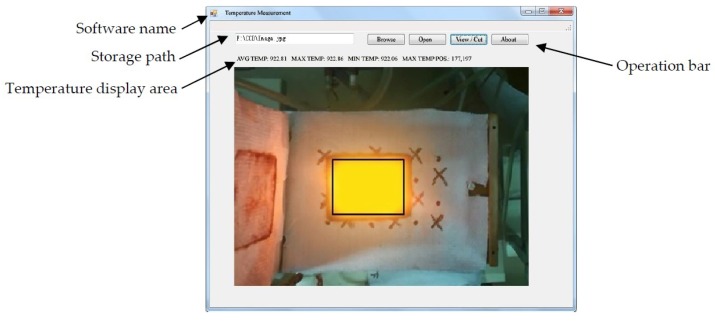
Temperature measurement software interface introduction.

A temperature test was carried out using B1500HS boron steel. The experimental plate was heated by an SP-25 induction heating device, and the temperature was measured with a Raynger 3i Plus temperature gun while photographed with a Canon EOS 80D camera under ISO-3200. Thus, the temperature was recorded in the temperature gun and the temperature measurement software with the image obtained in the camera at the same time. The software interface is shown in Figure 7 which contains the operation bar, the temperature display area, etc. The temperature results obtained in the software were compared with the data recorded in real-time monitoring of the temperature measuring gun, as shown in Table 2. The temperature measurement error was within 8 °C, which met the temperature measurement requirement of the B1500HS boron steel in electromagnetic induction heating treatment.

**Figure 8:**
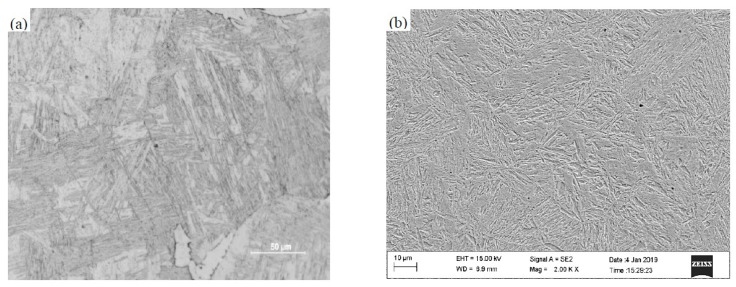
Metallograph of B1500HS after quenching in magnification of (**a**) 500 times and (**b**) 2000 times.

In order to further analyze the heating state of the steel plate, the microstructure after quenching was analyzed. The microstructure was observed by Leica DM2500M metallographic microscope and Ultra Plus field emission scanning electron microscope. As shown in Figure 8a,b, the microstructure was completely lath martensite, which indicated that the original ferrite and pearlite were all transformed into austenite. The heating temperature of the sample was higher than the complete austenitizing temperature of the sample, and the temperature measurement system met the experimental temperature measurement requirements.

## 5. Conclusions

The color CCD technology was used to analyze the heat radiation distribution of high temperature steel plates based on the special temperature field simulation.

1. Based on the theoretical analysis, the relevant functional modules of the temperature measurement system were developed. It realized functions such as image reading, image preprocessing, point temperature calculation, field average temperature calculation, field minimum and minimum value calculation and coordinate display.
2. Through error analysis and temperature calibration, the error of the temperature measurement system was acceptable and the error range was within 8 °C.
3. Through the B1500HS boron steel test of quenching by electromagnetic induction heating, the temperature measurement system was stable and met the experimental requirements. The temperature measurement system has a simple structure and strong practicability, and it could become an effective tool for non-contact measurement of high temperature steel plates after further development.

## Figures and Tables

**Table 1:** Error analysis of distance change.

Different Distances (mm)	Temperature Measurement Point (°C)						
800	840	850	860	870	880	890	900
400	840.7	853.4	862.2	867.8	882.5	888.6	903.0
600	839.1	853.7	863.1	872.5	882.4	891.8	899.4
1000	838.9	851.1	863.2	867.2	883.6	892.6	898.2
1200	842.3	852.2	857.4	873.0	883.3	893.5	902.1
Absolute deviation	2.3	3.6	3.2	3.0	3.6	3.5	3.0
Maximum standard deviation	1.6	2.6	2.3	2.1	2.6	2.4	2.1

**Table 2:** Temperature results comparison.

Measuring Method	Temperature Results (°C)				
Temperature measurement software system	910.6	915.3	922.8	929.3	931.1
Temperature measuring gun	902.9	910.7	918.4	922.6	925.2
